# Predicting Inter-Species Cross-Talk in Two-Component Signalling Systems

**DOI:** 10.1371/journal.pone.0037737

**Published:** 2012-05-22

**Authors:** Sonja Pawelczyk, Kathryn A. Scott, Rebecca Hamer, Gareth Blades, Charlotte M. Deane, George H. Wadhams

**Affiliations:** 1 Department of Biochemistry, Oxford Centre for Integrative Systems Biology, University of Oxford, Oxford, United Kingdom; 2 Department of Biochemistry, University of Oxford, Oxford, United Kingdom; 3 Department of Statistics, University of Oxford, Oxford, United Kingdom; Yale Medical School, United States of America

## Abstract

Phosphosignalling pathways are an attractive option for the synthetic biologist looking for a wide repertoire of modular components from which to build. We demonstrate that two-component systems can be used in synthetic biology. However, their potential is limited by the fact that host cells contain many of their own phosphosignalling pathways and these may interact with, and cross-talk to, the introduced synthetic components. In this paper we also demonstrate a simple bioinformatic tool that can help predict whether interspecies cross-talk between introduced and native two-component signalling pathways will occur and show both *in vitro* and *in vivo* that the predicted interactions do take place. The ability to predict potential cross-talk prior to designing and constructing novel pathways or choosing a host organism is essential for the promise that phosphosignalling components hold for synthetic biology to be realised.

## Introduction

Synthetic Biology aims at designing new or redesigning existing biological circuits for a particular purpose [1]–[2]. In most cases this is achieved by transplanting biological components (DNA or proteins) from one organism into a non-native host to implement the new or redesigned circuit within that host cell. An implicit condition for this is that the introduced components should function either independently to, or at least interact minimally and in a well characterised way with, the native host systems.

Many of the early circuits which have been created have used transcriptional activators and repressors to control gene expression in *Escherichia coli* [3]–[4]. However, the small number of these parts which are available for the synthetic biologist to use has forced many to look outside these traditional components and to start to try to implement pathways designed around phosphorylation based signalling, either using MAPK pathways in eukaryotic cells [5] or two-component signalling pathways in bacteria [6]. A potential limitation of these approaches is that the host cells contain many of their own phosphosignalling pathways and it is currently not possible to determine in advance whether any of these will interact with, and cross-talk to, the introduced synthetic components. Some cross-talk has been shown in the MAPK pathways for osmolarity and pheromone sensing in *Saccharomyces cerevisiae* where both pathways share a MAPKKK [7]–[8]. Inter-species cross-talk in bacterial systems has been shown from *Enterococcus faecium* to *Escherichia coli*, its sensor kinase VanS can activate the *E. coli* response regulator PhoB [9]. A recent study by Antunes et al. has shown possible cross-talk across kingdoms where heterologous expression of PhoB and OmpR from *E. coli* in *Arabidopsis* are phosphorylated by endogenous cytokinin-mediated HK-signaling components [10].

Here we focus on bacterial two-component systems (TCS) and develop and validate a method which allows potential cross-talk to be predicted prior to the design and implementation of a synthetic circuit in a particular host cell type. TCS are ubiquitous in bacteria and lower eukaryotes [11]. They are the main signal transduction pathways in these species and how an organism can coordinate the activity of so many highly related signalling systems is the focus of much attention [12]. TCS generally consist of a sensor histidine protein kinase (HPK) and a response regulator (RR). Classically HPKs contain a periplasmic sensory region, a transmembrane region and a cytoplasmic signalling region. The cytoplasmic part of the kinase consists of a HAMP linker domain (present in Histidine kinases, Adenyl cyclases, Methyl-accepting proteins and Phosphatases), a dimerisation and histidine phosphoaccepting domain (DHp) and a catatlytic domain (CA). The RR usually consists of a phosphyl receiver (REC) domain and a regulatory DNA binding domain.

In a canonical TCS the histidine kinase senses environmental changes which trigger autophosphorylation and phosphotransfer to the response regulator. This leads to a conformational change in the response regulator allowing binding to a specific region of DNA and regulation of transcription [13]–[14].

Despite the fact that a single bacterium can contain many tens or hundreds of TCS, they generally show a high degree of specificity with a single HPK phosphorylating only its partner RR [15]. Many studies have tried to dissect the specificity of these interactions to determine how the fidelity of signal transmission in maintained and unwanted cross-talk prevented when the HPK and RR families share such significant sequence and structural similarity [16]. It is generally thought that the majority, if not all, of the specificity information is contained within the DHp domain of the HPK and the REC domain of the RR [17].

Interestingly, cross-talk between non-native and host TCS components has been observed previously. When the vancomycin resistance TCS (VanS-VanR) from *Enterococcus faecilis* BM4147 was introduced into *E. coli* it was found to cross-talk to the native PhoR-PhoB system, but only when either the VanS or PhoR kinases were deleted [9]. In this case cross-talk was attributed to the elimination of the phosphatase activity usually associated with the cognate bifunctional HPK. Indeed, the presence of a bifunctional kinase/phosphatase for its cognate RR may be a general mechanism through which cross-talk is reduced *in vivo*. The presence of specific phosphatases may also offset any inappropriate phosphorylation of the RR by small molecule phosphodonors such as acetyl-phosphate [18]. *In vitro*, histidine kinases have been shown to phosphorylate a wide range of RRs, however many studies have shown that the HPK demonstrates a distinct kinetic preference for its natural *in vivo* RR [19].

One of the most intensively studied TCS is the EnvZ/OmpR system of *E. coli*. It is involved in sensing osmolarity changes in the environment and regulating the transcription of the outer membrane porins OmpC and OmpF [20] and consists of the osmosensing histidine kinase EnvZ and the response regulator OmpR. Under conditions of high osmolarity, EnvZ undergoes autophosphorylation on histidine 243 [21] and this phosphoryl group is then transferred to aspartic acid 55 on OmpR (to give OmpR∼P) [14]. Under low osmolarity conditions EnvZ acts as a phosphatase, converting OmpR∼P to OmpR. Phosphorylation of OmpR results in a conformational change, allowing it to bind to the promoter regions of either *ompC* or *ompF*, depending on the osmotic level of the cell’s environment. Under conditions of high osmolarity there are high concentrations of OmpR∼P and transcription of o*mpC* is preferred whereas under low osmolarity conditions there are low levels of OmR∼P and the transcription of *ompF* is favoured. [22]. In early attempts to redesign this system, the periplasmic ligand binding domain of the chemoreceptor Tar was fused with the cytoplasmic kinase/phosphatase domain of EnvZ, allowing the system to sense aspartate [23]–[25]. The resulting chimeric receptor, Taz, was able to activate OmpR in response to the extracellular levels of aspartate. In this paper we develop and validate a simple tool which assigns the likelihood of a given HPK interacting with particular RRs. We show that it can predict the presence of inter-species cross-talk when the non-native EnvZ/OmpR *E. coli* TCS components are introduced into a *Rhodobacter sphaeroides* host cell.

## Results

We have developed a simple analysis tool for the prediction of inter-species cross-talk between components of bacterial two-component systems. We created two non-redundant databases containing the DHp domains of the HPKs and the REC domains of the RRs from sequenced bacterial genomes respectively. The sequences were extracted from the Pfam database [26] and clustered using the program TribeMCL [27]. This resulted in 227 clusters for the DHp domains, each containing up to 1193 sequences from 466 species. For the REC domains of the RRs, 433 different clusters were created, containing up to 1191 sequences from 472 species. To predict interactions between members of the different clusters we used a statistical method to assign each of the DHp and REC domains to operons [28]. Using the same definition as the MiST2 database [29] DHp and REC domain containing proteins encoded in the same operon were considered to be probable interaction partners if there were no more than two intervening genes. From these data sets, we generated a probability table showing the likelihood that DHp domains from a particular cluster would interact with REC domains from other clusters. (Table S4).

As there is little experimental data on inter-species cross-talk, we validated our method by predicting intra-species partners for orphan HPKs. As these genes are orphans, ie not encoded in an operon with a RR, they will not have contributed to our interaction dataset. The *Caulobacter crescentus* orphans are the best studied experimentally and hence provide an ideal set to validate our method. We also compare our results to those obtained with two other recent methods for predicting partners for orphan kinases, those of Burger and van Nimwegen [19] and Procaccini et al. [20].

*C. crescentus* contains eight orphan HPKs. Table 1 shows the predicted RRs for each orphan HPK using our clustering method, the Procaccini method and the Burger method. Experimentally validated RRs are highlighted in green, experimentally disproved interactions in red and untested partners in black [19], [30]. Overall, the three methods gave similar results with our approach predicting a partner for seven of the orphans including four of those which have been experimentally validated. We did not predict the experimentally determined RR CenR as a partner for CenK, although as stated by Procaccini et al [20] CenK generates very weak scores with their method, indicating that these pairings are not yet well described by any of the current models. Interestingly, all three methods predict a different RR for the HPK CC_0586 and further experiments to determine which, if any, of these are correct would be informative.

**Table 1:** Comparison of response regulator predictions for *Caulobacter crecentus* histidine kinases.

orphan HK		Burger and vanNimwegen	Procacciniet al.	Pawelczyket al.
**DivL**	**CC_3484**	*DivK*/**CC_3477**	*DivK/PleD*	*DivK/PleD*
**PleC**	**CC_2482**	*DivK*/**CtrA**	*DivK/PleD*	*DivK/PleD*
**DivJ**	**CC_1063**	*PleD*/**CtrA**	*DivK/PleD*	*DivK/PleD*
**CenK**	**CC_0530**	*CenR*	*CenR*	no prediction
	**CC_2884**	DivK/PleD	DivK/PleD	DivK/PleD
	**CC_0586**	CC_3015	DivK	cluster 0
	**CC_2755**	CC_2757	CC_2757	CC_2757
	**CC_1062**	DivK/PleD	DivK/PleD	DivK

Comparison of response regulator predictions for *Caulobacter crecentus* histidine kinases. The table shows the predictions of possible response regulators for *C. crecentus* histidine kinases from different prediction tools. Experimentally validated RRs are highlighted in italic, experimentally disproved interactions in bold and untested partners in roman type.

### Prediction of Interspecies Cross-talk

We have chosen to introduce the well-characterised EnvZ/OmpR TCS from *E. coli* into an alternate host species. The host species chosen was *Rhodobacter sphaeroides* WS8N [31], a well-characterised model organism whose genomic DNA can be easily manipulated. We used our clustering approach to predict which of the native HPKs may cross-talk with the *E. coli* EnvZ/OmpR TCS. EnvZ appears in HPK cluster 11 (Figure S1). Interestingly, one of the *R. sphaeroides* HPKs, RSP0203, also appears in this cluster suggesting that it is a potential candidate for cross-talk (Table S2). The sequences of both proteins show no homology in their periplasmic domains, however the sequence similarity between the cytoplasmic regions (amino acid 163–450 for EnvZ, 161–493 for RSP0203) containing the HAMP, DHp and CA domains of EnvZ from *E. coli* and RSP0203 from *R. sphaeroides* is 36% according to the BLAST database [32].

RSP0203 is one of five *R. sphaeroides* orphan kinases, those HPKs whose corresponding RR is not apparent from genomic context. From our analysis of the interactions between clusters we would predict that kinases found in cluster 11 are most likely to interact with RRs in cluster 13 (Table S4). Interestingly, not only does OmpR appear in RR cluster 13, but also one of the *R. sphaeroides* orphan RRs, RSP1138, also appears in this cluster. We therefore predicted that the orphan *R. sphaeroides* HPK RSP0203 would phosphorylate the orphan RR RSP1138 and that cross-talk may occur between the HPK/RR pairing from *R. sphaeroides* and the EnvZ/OmpR system from *E. coli*.

According to the Pfam database, RSP0203 is a 439 amino acid protein containing a HAMP domain, a HisK A phosphoacceptor domain and a HATPase c domain and is therefore a classical HPK, similar in its domain structure to *E. coli’s* EnvZ. However, its putative signal sensing periplasmic domain shows no similarity to that of EnvZ or to any other protein in the database, has no identifiable domains and the whole protein is classified as an integral membrane sensor signal transduction histidine kinase in the BLAST database [33].

According to the Pfam database [26], RSP1138 is a 232 amino acid protein that contains an N-terminal CheY-like response regulator receiver domain and a C-terminal DNA binding domain in a helix-turn-helix motif. Both RSP0203 and RSP1138 are located on chromosome 1 in the genome of *R. sphaeroides* in position 1918334 bp (RSP0203) and 2901588 bp (RSP1138).

### *In vitro* Analysis of Interspecies Cross-talk

**Figure 1:**
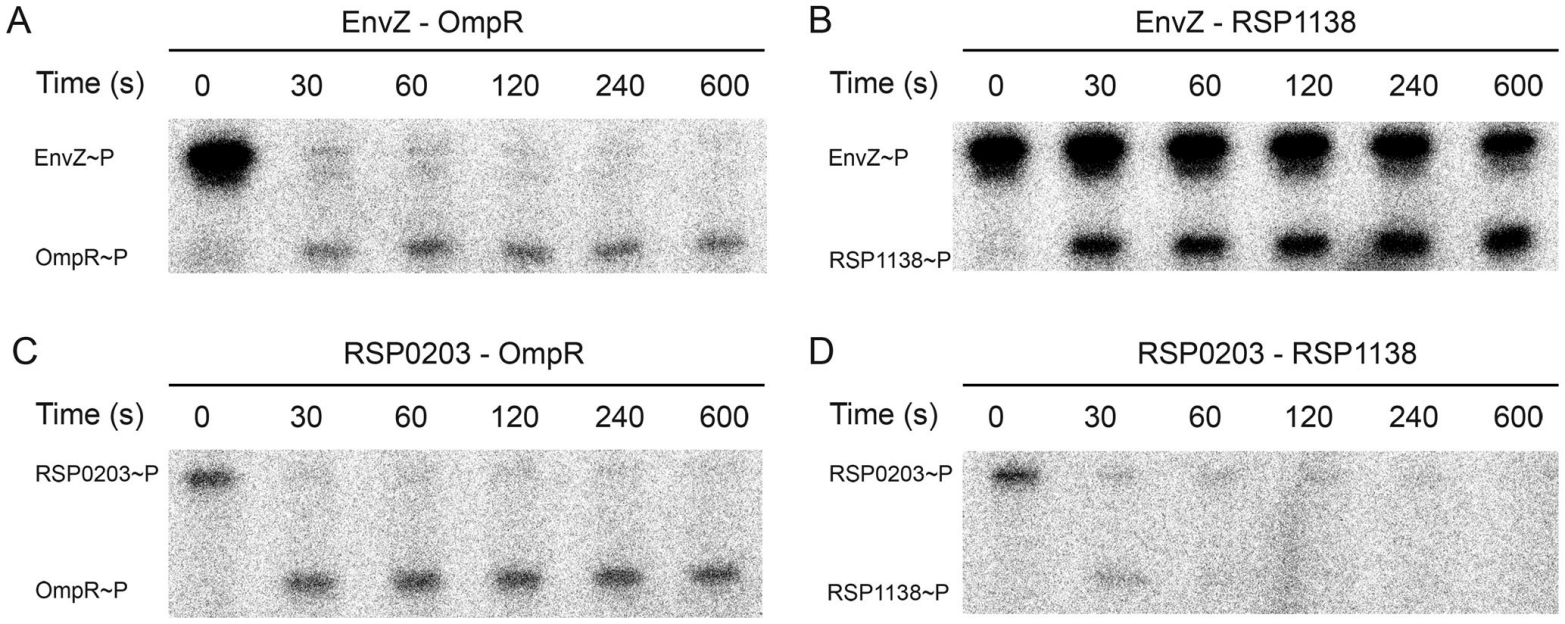
Phosphorylation assays of EnvZ/OmpR and RSP0203/RSP1138. Both histidine kinases were autophophorylated and tested for phosphotransfer to the response regulators after different time intervals. **A** Phosphotransfer from EnvZ to OmpR. **B** Phosphotransfer from EnvZ to RSP1138. **C** Phosphotransfer from RSP0203 to OmpR. **D** Phosphotransfer from RSP0203 to RSP1138.

The purified soluble cytoplasmic domains of the histidine protein kinases *E. coli* EnvZ and *R. sphaeroides* RSP0203 were analysed for their ability to phosphotransfer *in vitro* to the response regulators OmpR and RSP1138 (Figure 1). Both EnvZ and RSP0203 autophosphorylate and these assays clearly showed that phosphotransfer not only took place from EnvZ to its native response regulator OmpR and from RSP0203 to RSP1138, but also from the *R. sphaeroides* RSP0203 to OmpR and from *E. coli* EnvZ to *R. sphaeroides* RSP1138. The phosphotransfer assays also confirmed the observations of Yoshida et al. [34], that the kinase activity of EnvZ is dominant over its phosphatase activity for OmpR, and demonstrated that the same applies for RSP1138, under these conditions. The precise kinetics of phosphotransfer to the different response regulators may vary, as shown by the differing levels of phosphorylated EnvZ remaining at each timepoint (Figures 1A and 1B). The amount of phosphorylated kinase and RR present at each timepoint is dependent on the rate of phosphotransfer between kinase and RR and on the RR autodephosphorylation rate. One explanation for the difference in the result for OmpR and RSP1138 is that both phosphotransfer to and autodephosphorylation of the RR are enhanced for the cognate pair. However, determining the precise kinetics of these reactions is beyond the scope of this investigation.

### *In vivo* Analysis of Cross-talk

To determine whether RSP0203 can interact *in vivo* with the non-native response regulator OmpR from *E. coli* we designed a testbed where we introduced parts of the EnvZ/OmpR TCS along with a reporter construct into *R. sphaeroides* WS8N. The gene encoding CheA1, a chemotaxis protein not expressed under the chosen cultivation conditions [35], was replaced by the *E. coli* promoter of *ompC* translationally fused to the yellow fluorescence reporter gene *yfp* to generate strain JPA1800.

To test whether *E. coli* OmpR can interact with the native *R. sphaeroides* RNA polymerase and activate transcription, *R. sphaeroides* WS8N and JPA1800 were transformed with the inducible expression vector pIND4 [36] expressing either OmpR (pINDOmpR) or the non-phosphorylatable OmpRD55A (pINDD55A). Cells were grown under photoheterotrophic conditions and their fluorescence output data were measured by fluorometry (Table 2). In the absence of OmpR, no fluorescence output could be detected from JPA1800, demonstrating that no *R. sphaeroides* RR was able to bind and to induce expression from the promoter region of *ompC* in this reporter strain. The introduction of OmpR caused a significant increase in the fluorescence output, showing that the *E. coli* protein could not only bind to the promoter region of *ompC* in JPA1800 but also interact with the *R. sphaeroides* RNA polymerase. The absence of the cognate *E. coli* kinase EnvZ in JPA1800 suggested that a kinase from *R. sphaeroides* was responsible for the phosphorylation of OmpR in this strain, as only OmpR∼P is predicted to undergo the conformational change allowing it to bind to the promoter region of *ompC*. To verify that OmpR∼P was responsible for the activation of *yfp* transcription in JPA1800, we transformed this reporter strain with pINDD55A. This mutation of OmpR, the exchange of aspartic acid in position 55 to alanine, prevents its phosphorylation and hence activation [37]. The fluorescence output of the cells expressing OmpRD55A was comparable to wild-type (WS8N) level, confirming that only OmpR∼P was responsible for transcription activation of the reporter gene *yfp*.

**Table 2:** Fluorescence output data.

	Fluorescence outputper cell (a.u.)	standarddeviation
WS8N	59.05	6.52
WS8N pIND4	49.55	1.10
WS8N pINDOmpR	43.52	1.19
WS8N pINDD55A	53.10	1.65
JPA1800	68.45	1.24
JPA1800 pIND4	76.14	3.42
JPA1800 pINDOmpR	2191.93	23.01
JPA1800 pINDD55A	91.33	3.64

Fluorescence output data of wild type *R. sphaeroides* (WS8N) and the reporter strain *cheA1::PompC-yfp* (JPA1800) expressing OmpR or the non-phosphorylatable OmpRD55A Cells were grown under photosynthetic conditions for 72 h and analysed in a plate reader (Tecan, Austria). Transcription from the plasmids pIND4, pINDOmpR and pINDD55A was induced with 10 µM IPTG.

**Figure 2:**
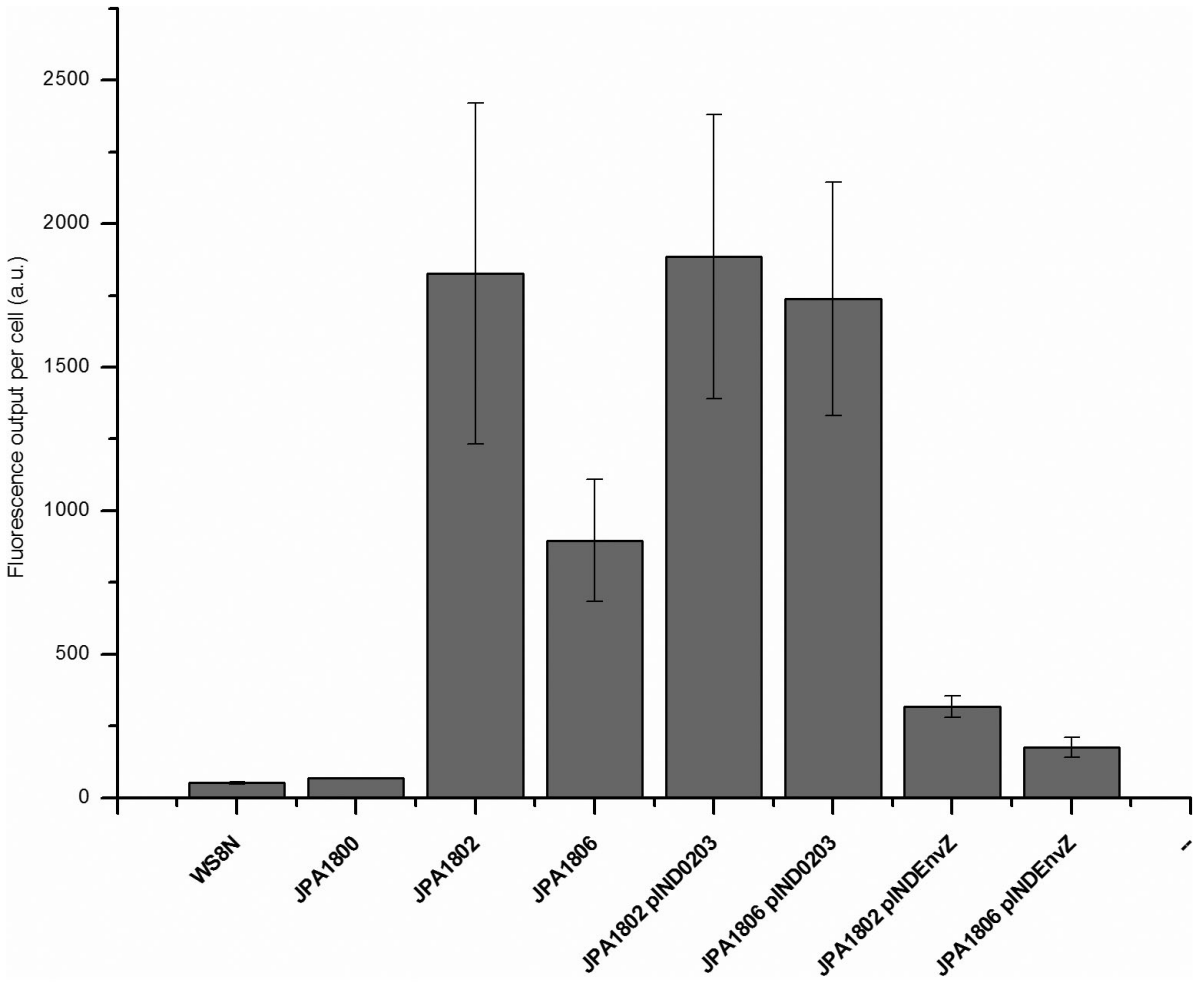
Fluorescence output data of *R. sphaeroides* and its derived mutants. Cells were grown under photosynthetic conditions for 72 h and analysed in a plate reader (Tecan, Austria). Transcription from the plasmids pINDEnvZ and pIND0203 was induced with 10 µM IPTG.

Further analysis of which kinase is responsible for the phosphorylation of OmpR *in vivo* required the introduction of the DNA sequence coding for OmpR into the *R. sphaeroides* genome. The gene encoding TlpC, a non-essential protein involved the chemotaxis signalling pathway [38], was replaced by that encoding the response regulator OmpR from *E. coli* in JPA1800 to generate JPA1802. The gene encoding TlpC was chosen for replacement as intracellular levels of expressed TlpC in *R. sphaeroides* are similar to expressed OmpR levels in *E. coli* [39] (M Gould, personal communication). The fluorescence output of JPA1802 clearly showed that OmpR was expressed in this strain, that it bound to the promoter region of *ompC* and that it initiated transcription of the reporter gene *yfp* (Figure 2).

These data (Figure 2, Table 3) showed that the *E. coli* OmpR must have been phosphorylated even in the absence of its cognate kinase (EnvZ) in *R. sphaeroides*, and from our previous analysis we predicted that the host kinase RSP0203 could be the source of this phosphorylation. The deletion of *rsp0203* from the genome of JPA1802, forming JPA1806, resulted in a marked reduction in the fluorescence reporter output compared to JPA1802 (Figure 2). This strongly suggested that RSP0203 is the major histidine kinase responsible for the phosphorylation of OmpR in *R. sphaeroides*.

**Table 3:** Table of strains and plasmids used in this study.

Strains or Plasmids	Description	Source/Reference
Bacterial Strains		
*Escherichia coli*W3110	F-, lambda-, IN(rrnD-rrnE)1, rph-1, general *E. coli* strain	[37]
S17-1λpir	Strain capable of mobilizing the suicide vector pK18mobsacB into *R. sphaeroides;* streptomycin-resistant	[38]
XL1 Blue	General cloning strain and expression host. *lacI^q^*; tetracycline resistance	Stratagene
M15pREP4	Expression host containing pREP4; kanamycin resistance	Qiagen
BL21	Expression host	NEB
*Rhodobacter* *sphaeroides* WS8N	Spontaneous nalidixic acid-resistant mutant of wild-type WS8	[27]
JPA1800	*cheA1::PompC-yfp,* derivative of WS8N	This study
JPA1802	*cheA1::PompC-yfp, tlpC::ompR*, derivative of WS8N	This study
JPA1803	ΔRSP0203, derivative of WS8N	This study
JPA1806	ΔRSP0203, *cheA1::PompC-yfp, tlpC::ompR*,derivative of WS8N	This study
Plasmids		
pEYFP-N1	Vector expressing the enhanced yellow fluorescence protein derived from *Aequorea victoria.*Confers ampicillin resistance	Clontech
pk18*mobsac*B	Allelic-exchange suicide vector mobilized by *E. coli* S17-1λpir. Confers kanamycin resistanceand sucrose sensitivity	[38]
pk18PompCyfp	Plasmid for allelic-exchange of the construct PompC-yfp in WS8N, derivative of pk18*mobsac*B	This study
pk18ompR	Plasmid for allelic-exchange of ompR in WS8N, derivative of pk18*mobsac*B	This study
pk18RSP0203	Plasmid for the deletion of RSP0203 in WS8N, derivative of pk18*mobsac*B	This study
pIND4	IPTG-inducible expression vector for *R. sphaeroides*. Confers kanamycin resistance	[31]
pINDEnvZ	Plasmid for expression of EnvZ in *R. sphaeroides*, derivative of pIND4	This study
pIND0203	Plasmid for expression of RSP0203 in *R. sphaeroides*, derivative of pIND4	This study
pQE60	IPTG-inducible expression vector for *E. coli*. Introduces RGS(H)6 at the C-terminus of theprotein. Confers ampicillin resistance	Qiagen
pQE60OmpR	Plasmid for the expression of HIS-tagged OmpR, derivative of pQE60	This study
pQE60RSP1138	Plasmid for the expression of HIS-tagged RSP1138, derivative of pQE60	This study
pQE60RSP0573	Plasmid for the expression of HIS-tagged RSP0573, derivative of pQE60	This study
pQE60RSP1083	Plasmid for the expression of HIS-tagged RSP1083, derivative of pQE60	This study
pQE60RSP2599	Plasmid for the expression of HIS-tagged RSP2599, derivative of pQE60	This study
pREP4	Plasmid containing the *lacIq* gene and conferring kanamycin resistance. Compatible with pQE60	This study
pGEX-6p-1	IPTG inducible expression vector. Introduces a GST tag at the N terminus of the expressed protein.Confers ampicillin resistance	GE Life Science
pGEXRSP0203	Plasmid for expression of the GST-tagged cyoplasmic region of RSP0203, derivative of pGEX-6p-1	This study
pGEXRSP2915	Plasmid for expression of the GST-tagged cyoplasmic region of RSP2915, derivative of pGEX-6p-1	This study
pGEXEnvZ	Plasmid for expression of the GST-tagged cyoplasmic region of EnvZ, derivative of pGEX-6p-1	This study

To confirm that RSP0203 was the major kinase phosphorylating OmpR in *R. sphaeroides*, RSP0203 was overexpressed from pIND4 and complementation of the RSP0203 deletion in JPA1806 restored wild-type levels of fluorescence (Figure 2). Interestingly, the overexpression of RSP0203 from pIND4 in JPA1802 did not increase the observed fluorescence signal. This suggests that sufficient OmpR∼P has already been produced in JPA1802 to saturate the single *ompC* promoter in this strain. The deletion of *rsp0203* did not completely abolish the fluorescence output of the reporter strain JPA1806, which could be explained either if OmpR is also being phosphorylated by a small molecule phosphodonor such as acetyl phosphate [18] or by another unidentified HPK.

The gene encoding the native histidine kinase of OmpR, EnvZ, was also cloned into pIND4. This plasmid, pINDEnvZ, was expressed in the *R. sphaeroides* strains JPA1802 and JPA1806. This resulted in an obvious reduction of the fluorescence output of the reporter strains JPA1802 and JPA1806 (Figure 2), suggesting that the phosphatase activity of EnvZ dominated over its kinase activity under these conditions in *R. sphaeroides* and reduced transcription from the *ompC* promoter to close to wild-type levels [22].

## Discussion

Synthetic biologists are constantly looking to increase the available parts lists from which they can design and construct novel pathways and phosphosignalling systems. Both bacterial two-component systems and eukaryotic MAPK pathways provide a plethora of opportunities. However, with the increased diversity of parts comes the problem of the number of potential host systems with which the synthetic systems could interact. The ability to predict potential cross-talk prior to designing and constructing novel pathways or choosing a host organism is essential for the promise that phosphosignalling components hold for synthetic biology to be realised.

In this study, we demonstrate that it is possible to introduce parts of TCS into non-host strains for use as synthetic biology components. We show that the a non-host transcription factor, in this case OmpR from the γ-subgroup bacterium *E. coli*, is capable of binding the α-subgroup bacterial host’s (*R. sphaeroides*) RNA polymerase to activate transcription of a reporter construct. We also demonstrate that a host kinase phosphorylated this non-native RR *in vivo* and hence that the potential issue of cross-talk is a real problem with the use of these systems.

Most, if not all, of the specificity of HPK/RR interactions is encoded in the DHp and REC domains [19]. We therefore extracted the sequences of these domains from available protein sequences and clustered them by their sequence similarity. Using no additional information other than genome context we calculated the frequency with which HPKs of a particular cluster interacted with members of RR clusters across 466 species. Using our prediction tool, we identified a native *R. sphaeroides* kinase (RSP0203) as the probable cause of cross-talk to OmpR. The *in vitro* analysis of cross-talk between EnvZ/OmpR and RSP0203 demonstrated that predicted interactions between proteins within the clusters generated by our analysis do occur as we showed that both these kinases from the same cluster phosphotransfer to the response regulator OmpR.

Deletion of *rsp0203* from the JPA1802 reporter strain reduced the fluorescence expression levels, indicating *in vivo* that the predicted kinase RSP0203 was responsible for significant phosphorylation of the non-native RR OmpR. When the *E. coli* bifunctional kinase EnvZ was introduced into the *R. sphaeroides* reporter strains on a plasmid, a clear reduction of the fluorescence output was detectable, suggesting that the phosphatase activity of the native cognate partner protein exceeded the kinase activity of the predicted host’s cross-talking histidine kinase RSP0203. Unfortunately, experimental techniques for quantifying the specific kinase/phosphatase kinetics *in vivo* are not currently available, however further investigation of this observation are ongoing. The prediction of possible cross-talk between TCS is essential for our ability to utilise non-native biological components in the pursuit of synthetic biology.

A synthetic biologist proposing to use TCS proteins will have to consider the problem of cross-talk from host proteins. Our predictive tool can easily be used to provide guidance as to which (if any) of the host proteins would cross-talk to the synthetic components prior to the construction of synthetic strains. Assuming that both the bacterium from which the non-native components will be taken and the host are already contained within our database then simply searching the HPK or RR data tables for the GI number of the protein to be introduced will provide the cluster to which it has been assigned. These clusters can then be sorted by species and checked for the presence of any potential cross-talking host proteins. Should the protein to be introduced come from a bacterium which is not contained within our database, then these DHp or REC domain sequence can be compared against our dataset using a local BLAST search to find the HPK or RR cluster containing the most similar sequences (Figure S1, Methods S1, Tables S1, S2, S3 and S4) and a similar procedure followed.

Finally, our predictive tool could be used by researchers looking for the probable interaction partners of orphan HPKs and RRs. The probability table provides information about the most likely interaction clusters for a given orphan HPK or RR and may guide the search for the cognate pairings for these proteins. As was demonstrated by our *in vitro* phosphotransfer data, the orphan HKP RSP0203 was shown to phosphotransfer to the orphan RR RSP1138 as predicted from their cluster locations.

Our method also performed well in predicting the RRs for the orphan kinases of *C. crescentus.* Our predictions were in good general agreement with the experimental data [16], [30] and with predictions obtained by two other methods, those of Burger and van Nimwegen [19] and Procaccini et al [20]. The Burger method utilises a Bayesian approach to match orphan HPKs to RRs whereas the Procaccini method uses Direct-Coupling Analysis to extract residue-residue contacts and generates a scoring function to aid in the prediction of interaction partners. All three methods are well suited to the study of bacterial two component systems. For more general protein interaction predictions, a distinct advantage of the Burger method is that it can be applied where no large set of interaction partners is known, whereas the Procaccini method provides a direct scoring function as its output. As with the Procaccini method, ours requires a large dataset and also assumes a one to one relationship between HPK and RR which whilst generally true for TCS may not always be the case. The clear advantage of our method is simplicity, using no more information than the sequences and genome context information to generate the clusters and that it provides a simple analysis that predicts inter-species cross-talk across a wide range of potential host organism. For all prediction models the scarcity of experimental data somewhat hinders their validation. However, from the existing data in the literature [16], [30] and the analysis of the representatives of the clusters tested here our method appears to provide a good guide for researchers working in this area. Further validation of the method using members of other clusters is currently underway. Whilst most predictions were the same regardless of the method used, in the case of CC_0586 the three methods all predicted different RRs. This suggests that the ability for researchers to apply a combination of different methods for orphan protein interaction predictions will undoubtedly aid the experimental design in this area.

In summary, our results demonstrate the importance of considering and being able to predict how introduced synthetic biological components will interact with those of the native host cell. We have provided a simple bioinformatic tool to allow synthetic biologists to predict whether interspecies cross-talk between introduced and native two-component signalling pathways will occur and shown both *in vitro* and *in vivo* that the predicted interactions do take place. We have also shown that our prediction tool can aid in the identification of response regulators for orphan kinases.

## Materials and Methods

### Bioinformatics

#### Histidine kinases and response regulators

A set of bacterial protein sequences from 504 non-redundant species were downloaded (February 2009) from the NCBI (ftp://ftp.ncbi.nih.gov/genomes/Bacteria, multiple strains of the same species were not included). HMMER version 3.0 ([40], http://hmmer.janelia.org) was used to find response regulator REC domains and DHp domains of histidine kinases. The PFAM hidden markov models for REC domains (PF00512; ([26]) and for DHp domains (PF00512) were employed.

**Table 4:** Table of primers used in this study.

Primer	Sequence
TLPCup SalI	5′-GGCCAGGTCGACCCGATCGTGATCGTCCAGC-3′
TLPCup OE	5′-GTAGTTCTCTTGCATCGGACGGTCCTTTCCGTG-3′
OmpR OE	5′-CACGGAAAGGACCGTCCGATGCAAGAGAACTAC-3′
OmpR XbaI	5′-CGATAGTCTAGATCATGCTTTAGAGCCGTCCG-3′
TLPCdwn XbaI	5′-CGATAGTCTAGA CCCTTGCCCCCTGCTTC-3′
TLPCdwn SphI	5′-CTAGCGCATGC CTCGGCCGACTCATTGCTG-3′
CheA1up FW EcoRI	5′-CGTACGGAATTCCCTGGCGGCCGGACAGGTC-3′
CheA1up RV	5′-GGACGAGCTGTACAAGTAAGGTGGCCTCACGCG-3′
PompC-YFP BamHI	5′-AGCCGCGGATCCTTGACATTCAGTGCTGTC-3′
ompC-YFP RV OE	5′-GCCCTTGCTCACCATGAACTGGTAAACCAG-3′
PompC-YFPFW	5′-CGCGTGAGGCCACCTTACTTGTACAGCTCG-3′
ompC-YFP FW OE	5′-CTGGTTTACCAGTTCATGGTGAGCAAGGGC-3′
CheA1dwn BamHI	5′-AGCCGCGGATCCGATGGCGGCCGTGAGCCTGG-3′
CheA1dwn SalI	5′-GTCCGCGTCGACGGGTCACGGACTGCAGTCGG-3′
Δ0203up FW MfeI	5′-GATCGCCAATTG TCCCCGTCCGGAGGAGAGAC-3′
Δ0203up RV BamHI	5′-CATTGCGGATTCGGGCCGACCCTAGCCCTGCG-3′
Δ0203Dwn FW BamHI	5′-CATTGCGGATTCTCCGGCGGCTGGTCCTTTCG-3′
Δ0203dwn RV SalI	5′-CAGATCGTCGACCTCCGGCAACACGCCCGCCC-3′
pINDEnvZ FW BspHI	5′-GCCGATTCATGAGGCGATTGCGCTTCTC-3′
pINDEnvZ RV BamHI	5′-AGCCGCGGATCCTTACCCTTCTTTTGTCGTGCCCTGCG-3′
pIND0203 FW AflIII	5′-ACTGAC ACATGT TCGCGCCGCTCAAATCCTTCGT-3′
pIND0203 RV BglII	5′-ATCAGTAGATCTTCAGCGGGCGAGCGTCAGTT-3′
pGEXEnvZ FW BamHI	5′-AGCCGCGGATCCCGGCTGGTGTTAAGC-3′
pGEXEnvZ RV XhoI	5′-AGCCGCCTCGAGCCCTTCTTTTGTCGTGC-3′
pGEXRSP0203 FW EcoRI	5′-ACGTACGAATTCCGTTCCCGGATCGAG-3′
pGEXRSP0203 RV SalI	5′-ATCAGTGTCGACGCGGGCGAGCGTCAG-3′
pQE60ompR FW BsaI	5′-ACGCGATGGTCTCACATGCAAGAGAACTACAAG-3′
pQE60ompR RVBamHI	5′-AGCCGCGGATCCTGCTTTAGAGCCGTC-3′
pQE601138 FW BspHI	5′-CCTGATTCATGAGTGAGCCACAGGCGCATC-3′
pQE601138 RVBamHI	5′-AGCCGCGGATCCGGGCTACATGCTCGCGCC-3′

16742 proteins were found to have at least 1 REC domain, where the e value of the hit was below the default inclusion threshold of 0.01. The sequences of all REC domains were extracted from these proteins. As some proteins had more than one REC domain, this gave 17448 sequences. 9881 proteins were found to have at least 1 DHp domain, where the e value of the hit was below the default inclusion threshold of 0.01. The sequences of the DHp domains were extracted from these proteins. As some proteins had more than one DHp domain, this resulted in 9906 sequences. All heuristic filters were turned off to ensure maximum sensitivity.

#### Clustering

BLAST version 2.2.23 [33], [41] was used to compare all 9881 HK DHp domains to each other. DHp sequences were then clustered using TRIBE-MCL [27]. The same was done for the 17448 REC domain sequences. The I value for the clustering was 5.

### Molecular Biology

#### Construction of *R. sphaeroides* mutant strains

For all strains, plasmids and primers used in this study, please see Table 3 and Table 4. A transcriptional fusion of *yfp* (pEYFP-N1, Clontech) to the promoter region (300 bp upstream of *ompC*) of *ompC* from *E. coli* W3110 [42] flanked by the 500 bp up- and down-stream region of *cheA1* (*Eco*RI, *Sal*I) was cloned into pK18*mobsacB* [43], linearised with *Eco*RI and *Sal*I, to create pK18ompCyfp. For the second mutation *E. coli* W3110 *ompR* (*Sal*I, *Xba*I) flanked by the 500 bp up- and downstream region of the *R. sphaeroides tlpC* was cloned into pK18*mobsacB,* linearised with *Sal*I and *Xba*I, resulting in pK18ompR. The constructs were sequenced to ensure that the regions were in frame and contained no errors. The mutations were introduced into the genome of *Rhodobacter sphaeroides* WS8N [31] by allelic exchange as described previously [43]–[44]. The correct insertions were confirmed by Southern Blot. This resulted in the *R. sphaeroides* mutants JPA1800 (*cheA1::PompC-yfp*) and JPA1802 (*cheA1::PompC-yfp, tlpC::ompR*).

For the deletion of RSP0203 the 600 bp up- and down-stream regions of *rsp0203* were amplified by PCR including suitable restriction sites (*MfeI, SalI*) and were spliced together using overlap-extension PCR. The resulting fragment was cloned into *Mfe*I and *Sal*I linearised pK18*mobsacB* to create pK18RSP0203. The mutations were introduced into the genome of *Rhodobacter sphaeroides* WS8N and JPA1802 as described above. This resulted in the strains JPA1803 and JPA1806.The correct insertions were confirmed by Southern Blot.

#### Construction of plasmids

For the expression of EnvZ and RSP0203 in *R. sphaeroides,* the full length sequences of *E. coli* W3110 EnvZ and *R. sphaeroides* RSP0203 were cloned into the expression vector pIND4, resulting in pIndEnvZ and pIND0203. These plasmids were introduced to *R. sphaeroides* as previously described [36].

The full length sequences of wild-type *E.coli ompR* and wild-type *R. sphaeroides rsp1138* were amplified by PCR and cloned into linearised pQE60 (Qiagen) for the expression of HIS-tagged proteins.

The coding cytoplasmic sequences of *R. sphaeroides rsp0203* and *E.coli envZ* were amplified by PCR and cloned into the expression vector pGEX (GE Healthcare).

#### Purification of HIS/GST-tagged proteins

The HIS- and GST-tagged proteins were overexpressed and purified as described previously [45]–[46]. Cells containing the appropriate expression plasmids were grown in 2YT medium with antibiotics as appropriate to an OD600 of 0.8 at 37°C. Expression was induced by the addition of 0.1 mM IPTG for 20 h at 18°C. After induction, cells were harvested by centrifugation and resuspended in lysis buffer. Cells were lysed and filtered through a 0.45-µm (pore-size) syringe filter. The filtered supernatant was applied to a Ni-nitrilotriacetic acid agarose column (Qiagen), for HIS-tagged proteins, or a glutathione sepharose column (GE Healthcare), for GST-tagged proteins, equilibrated with lysis buffer. The column was washed lysis buffer prior to elution of the protein in lysis buffer supplemented with 250 mM imidazole (HIS-tagged proteins) or 10 mM reduced glutathione (GST-tagged proteins) (Figure S2).

#### Fluorescence assay

Cells were plated on solid LB containing naldixic acid and kanamycin, if needed. The plates were incubated at 30°C for 48 h. Single colonies were transferred into succinate medium and incubated at 30°C for 48 h in the light to reach stationary phase.

To obtain the fluorescence (wavelength YFP: excitation 508 nm, emission 540 nm; wavelength CFP: excitation 430 nm, emission 480 nm; gain: 99; flashes: 25) and optical density (OD; absorbance: 700 nm) readings from the cells 100 µl of stationary phase cultures were applied to each well of a black clear bottom 96-well plate (Corning) and measured in a plate reader (Tecan, Infinite M200). To calculate the fluorescence intensity per cell, the fluorescence output data were divided by the OD.

#### Phosphotransfer assays

Phosphotransfer assays of RSP0203 and EnvZ to OmpR were performed at 20°C in TGMNKD buffer (50 mM Tris HCl, 10% (v/v) glycerol, 5 mM MgCl_2_, 150 mM NaCl, 50 mM KCl, 1 mM DTT, pH 8.0) as previously described [47]. Reactions were initiated by the addition of 0.5 mM [γ-^32^P] ATP (specific activity 14.8 GBq mmol^-1^; PerkinElmer). The ATP-dependent phosphorylation of 5 µM RSP0203 and EnvZ was allowed to proceed for 30 min and then the phosphotransfer reactions were initiated by the addition of 20 µM response regulator. After 30 s, 60 s, 120 s, 240 s and 600 s a 10 µl aliquot of the reaction mixture was taken, quenched immediately in 10 µl of 3 x SDS-PAGE loading dye (7.5% (w/v) SDS, 90 mM EDTA, 37.5 mM Tris HCl, 37.5% glycerol, 3% (v/v) β-mercaptoethanol, pH 6.8) and analysed by SDS-PAGE (15% (w/v) acrylamide)).

To minimize hydrolysis of phosphoproteins, electrophoresis was performed at 4°C, with a run time of 90 minutes. Gels were exposed to phosphor screens (Kodak) and analyzed using an SF-phosphorimager with ImageQuant software (version 5.0, Molecular Dynamics) [48].

## Supporting Information

Figure S1 **Flow diagram explaining the finding of potential interaction partners for orphan proteins.** The diagram shows how a potential cognate partner for the orphan kinase RSP0203 from *R. sphaeroides* was found by applying our described method.
(DOC)

Figure S2 **Coomassie-stained protein gel.** Protein gel (RunBlue 4–20% precast gel, expedeon) of the purified GST-tagged cytoplasmic region of RSP0203 (56 kDa), the purified GST-tagged cytoplasmic region of EnvZ (57 kDa), HIS-tagged RSP1138 (28 kDA)and HIS-tagged OmpR (27 kDa).
(DOC)

Table S1 **Index of spreadsheet headings.**
(DOC)

Table S2 **Histidine Kinases.**
(XLS)

Table S3 **Response Regulators.**
(XLS)

Table S4 **Probability.**
(XLS)

Methods S1 **Finding potential interaction partners.**
(DOC)
