# Supplementary material for: Predicting Inter-Species Cross-Talk in Two-Component Signalling Systems
Source: PLoS One. 2012 May 22;7(5):e37737. doi: 10.1371/journal.pone.0037737 (PMC3358273; doi:10.1371/journal.pone.0037737)
Supplement: Figure S1 — Flow diagram explaining the finding of potential interaction partners for orphan proteins. The diagram shows how a potential cognate partner for the orphan kinase RSP0203 from R. sphaeroides was found by applying our described method. (DOC) [file pone.0037737.s001.doc]

**Figure S1: Flow diagram of finding potential interaction partners**

Identify your kinase of interest by GI number from column #GI in “Table S2”

e.g. for RSP0203 this is GI 77463771

Obtain histidine kinase cluster number from column “Cluster” in “Table S2”

Open “Table S4” and look in column “# HK_Index” for cluster 13.

e.g. for RSP0203 this is cluster 13

Find the response regulator cluster number with the highest probability of interaction. This will be judged by three values:

1. “Frequency” – Number of known interaction of proteins from HK and RR cluster
2. “Percentage_HK”- Percentage of HKs from cluster interacting with RR from this cluster
3. “Percentage_RR”- Percentage of RRs from cluster interacting with HK from this cluster

e.g. RR cluster 11 is Frequency: 107, Percentage_HK: 89.17, Percentage_RR: 72.3

Sort “Table S3” by column “Organism” and look up cluster 11

Cluster 11 contains one RR from *R. sphaeroides* (RSP1138)

Figure S1: Flow diagram explaining the finding of potential interaction partners for orphan proteins. The diagram shows how a potential cognate partner for the orphan kinase RSP0203 from *R. sphaeroides* was found by applying our described method.
