# Supplementary material for: Predicting Inter-Species Cross-Talk in Two-Component Signalling Systems
Source: PLoS One. 2012 May 22;7(5):e37737. doi: 10.1371/journal.pone.0037737 (PMC3358273; doi:10.1371/journal.pone.0037737)
Supplement: Figure S2 — Coomassie-stained protein gel. Protein gel (RunBlue 4–20% precast gel, expedeon) of the purified GST-tagged cytoplasmic region of RSP0203 (56 kDa), the purified GST-tagged cytoplasmic region of EnvZ (57 kDa), HIS-tagged RSP1138 (28 kDA)and HIS-tagged OmpR (27 kDa). (DOC) [file pone.0037737.s002.doc]

Figure S2: Coomassie-stained protein gel

Figure S2: Coomassie-stained protein gel. Protein gel (RunBlue 4-20% precast gel, expedeon) of the purified GST-tagged cytoplasmic region of RSP0203 (56 kDa), the purified GST-tagged cytoplasmic region of EnvZ (57 kDa), HIS-tagged RSP1138 (28 kDA)and HIS-tagged OmpR (27 kDa).
