# Supplementary material for: Predicting Inter-Species Cross-Talk in Two-Component Signalling Systems
Source: PLoS One. 2012 May 22;7(5):e37737. doi: 10.1371/journal.pone.0037737 (PMC3358273; doi:10.1371/journal.pone.0037737)
Supplement: Table S1 — Index of spreadsheet headings. (DOC) [file pone.0037737.s003.doc]

**Table S1: Index of spreadsheet headings**
