# Supplementary material for: Predicting Inter-Species Cross-Talk in Two-Component Signalling Systems
Source: PLoS One. 2012 May 22;7(5):e37737. doi: 10.1371/journal.pone.0037737 (PMC3358273; doi:10.1371/journal.pone.0037737)
Supplement: Methods S1 — Finding potential interaction partners. (DOC) [file pone.0037737.s007.doc]

**Methods S1 :Finding potential interaction partners**

In order to find a potential interaction partner for the user’s protein of interest from our datasets, one have to check the attached Excel files for the presence of that protein. If the protein of interest, HK or RR, is present in the file (Histidine Kinases.xls or Response Regulators.xls) note it’s assigned cluster number. In a separate Excel file, Probability.xls, the calculated probability of cluster interaction is listed. Find the cluster number of interest and therefore the most likely interaction cluster (see flow diagram below) If the protein of interest cannot be found in the datasets (Histidine Kinases.xls or Response Regulators.xls) the following procedure can be of use. This includes the protein of interest to be blasted against the used Pfam datasets for the histidine kinases or response regulators.

1. Download and install BLAST on your own computer – see:

**http://blast.ncbi.nlm.nih.gov/Blast.cgi?CMD=Web&PAGE_TYPE=BlastDocs&DOC_TYPE=Download**

for information.

Files can be downloaded from:

**ftp://ftp.ncbi.nih.gov/blast/executables/blast+/LATEST/**

- 1. There is no Graphical User Interface for blast. On windows commands are typed into a command prompt window. This window can be opened by clicking on **"Start
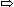
Program
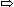
Accessories
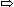
Command Prompt"** or by clicking **"Start
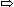
Run,"** typing **cmd** and pressing enter.

1. Download the HK file of protein sequences we provide in fasta format, PF00512_HisKA_whole_seqs.fasta.tar.gz
2. Uncompress the fasta file (e.g. on linux you would type:

tar –xvf PF00512_HisKA_whole_seqs.fasta

on Windows use e.g. Winzip.

http://www.gzip.org/ has more information on extracting .gz files).

1. Format the fasta file into a blastable database using makeblastdb. For example, on windows, to format the fasta file C:\Users\MyUser\Project\Database\PF00512_HisKA_whole_seqs.fasta type in the command prompt window:

“makeblastdb –in C:\Users\MyUser\Project\Database\PF00512_HisKA_whole_seqs.fasta

–dbtype prot”

1. Blast this database using your protein sequence (in fasta format) as a query. For example, on windows, to blast the test file C:\Users\MyUser\Project\MyProtein.fasta against the database C:\Users\MyUser\Project\Database\ PF00512_HisKA_whole_seqs.fasta and have the output file C:\Users\MyUser\Project\Results.blast type in the command prompt window type:

“blastp –query C:\Users\MyUser\Project\MyProtein.fasta –db C:\Users\MyUser\Project\Database\PF00512_HisKA_whole_seqs.fasta –out C:\Users\MyUser\Project\Results.blast”
